# Kin selection, quorum sensing and virulence in pathogenic bacteria

**DOI:** 10.1098/rspb.2012.0843

**Published:** 2012-05-30

**Authors:** Kendra P. Rumbaugh, Urvish Trivedi, Chase Watters, Maxwell N. Burton-Chellew, Stephen P. Diggle, Stuart A. West

**Affiliations:** 1 Department of Surgery, Texas Tech University Health Sciences Center, Lubbock, TX 79430, USA; 2 Department of Zoology, University of Oxford, Oxford OX1 3PS, UK; 3 School of Molecular Medical Sciences, Centre for Biomolecular Sciences, University of Nottingham, University Park, Nottingham NG7 2RD, UK

**Keywords:** communication, inclusive fitness, signalling, cheat

## Abstract

Bacterial growth and virulence often depends upon the cooperative release of extracellular factors excreted in response to quorum sensing (QS). We carried out an *in vivo* selection experiment in mice to examine how QS evolves in response to variation in relatedness (strain diversity), and the consequences for virulence. We started our experiment with two bacterial strains: a wild-type that both produces and responds to QS signal molecules, and a *lasR* (signal-blind) mutant that does not release extracellular factors in response to signal. We found that: (i) QS leads to greater growth within hosts; (ii) high relatedness favours the QS wild-type; and (iii) low relatedness favours the *lasR* mutant. Relatedness matters in our experiment because, at relatively low relatedness, the *lasR* mutant is able to exploit the extracellular factors produced by the cells that respond to QS, and hence increase in frequency. Furthermore, our results suggest that because a higher relatedness favours cooperative QS, and hence leads to higher growth, this will also lead to a higher virulence, giving a relationship between relatedness and virulence that is in the opposite direction to that usually predicted by virulence theory.

## 1. Introduction

The growth and virulence of pathogenic bacteria often depends on the cooperative production of extracellular factors, which are released in response to a cell-to-cell signalling process that has been termed quorum sensing (QS) [1,2]. Cells release small diffusible signal molecules, which have two effects. First, their uptake stimulates the release of extracellular factors, such as enzymes and nutrient-scavenging molecules, which facilitate growth. Second, their uptake stimulates the production of more signal molecules in a process that has been termed autoinduction. This leads to a positive feedback at high cell densities, which markedly increases production of extracellular factors [2]. The idea here is that the production of extracellular factors will be most beneficial at high cell densities, and that QS provides a way to coordinate this [3].

Theory predicts that the evolutionary stability of QS should depend upon the relatedness between interacting cells, and therefore the number of strains infecting each host [4]. The problem with producing extracellular factors is that cells could be exploited by ‘free-riders’ or cheats, who avoid the costs of producing QS-regulated factors themselves, but are able to benefit from those produced by others [4–6]. Genetic relatedness provides a solution to this problem, because the benefits will then be shared with individuals who share the genes for cooperatively producing extracellular factors [7]. However, while this kin selection explanation is appealing for QS and a range of other microbial social traits, there is a lack of direct empirical tests [8–10]. Furthermore, most empirical support for the application of kin selection to such microbial cooperation and signalling has come from the relatively unnatural environment of a liquid culture in a test tube [11–13], and it is not clear that cells will interact to the same extent under more natural conditions, such as during infection [14–16].

Here, we examine bacterial infections of mice, to test how relatedness influences selection for QS, with an experimental evolution approach. We study *Pseudomonas aeruginosa*, an opportunistic pathogen of plants and animals, including humans [17,18]. We used two bacterial strains: a wild-type that both produces and responds to QS signal molecules, and a *lasR* (signal-blind) mutant that does not respond to signal [16]. The *lasR* mutant represents a ‘free-rider’ that does not produce any QS-regulated extracellular factors. We started our experiment with a 50 : 50 mixture of these two strains, and then maintained them under conditions of either relatively low or relatively high relatedness. Our prediction is that the wild-type will be favoured under conditions of high relatedness, whereas the *lasR* mutant will be favoured under conditions of low relatedness. In addition, we test our underlying assumption that the wild-type is better able to proliferate in mice than the *lasR* mutant.

## 2. Methods

### (a) Bacterial strains and acute wound mouse model

We used two strains of *P. aeruginosa*: PA14 (which is a commonly used, fully virulent wild-type strain) and a PA14 *lasR* mutant strain (PA14::*lasR*), which does not respond to signal molecule [16]. We used female Swiss Webster mice (Charles Rivers Laboratories, Wilmington, MA) that were 8–10 weeks old and weighed approximately 20 g. Before infecting with bacteria, we anesthetized the mice and then shaved their backs before administering a dorsal, full-thickness scald burn that covered approximately 15 per cent of their total body surface. Directly after burning, we infected the wound with 10^2^ colony-forming units (CFU) of *P. aeruginosa*, injected subcutaneously at the wound site. We allowed the bacteria to grow in the infected mice for 24 h, after which we euthanized the mice, excised the wound tissue and removed their livers. We then homogenized all of the tissue in sterile phosphate buffer saline (PBS) and pooled the homogenates. We then serially diluted the pooled homogenates, and plated them onto *Pseudomonas* isolation agar (PIA), so that we could obtain single colonies for counting.

### (b) *In vivo* selection experiment

**Figure 1.**
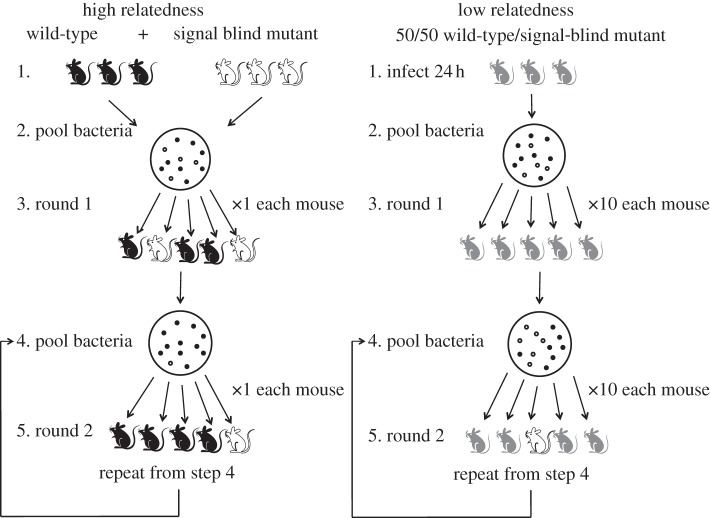
Experimental design. We varied relatedness by infecting each mouse (subpopulation) with either one clone (relatively high relatedness) or 10 (relatively low relatedness) clones. We use black to symbolize mice infected with the normal QS wild-type (PA14), white to symbolize mice infected with the mutant that does not respond to signal (PA14::*lasR*), and grey to symbolize mice infected with a mixture of these two types.

Our selection experiment contained two treatments: relatively high and relatively low relatedness (figure 1). The variation in relatedness in our experiment is with respect to QS, which is the trait in whose evolution we are interested. We are able to focus on QS because the wild-type and the *lasR* mutant are initially identical at other parts of the genome, and so there is no genetic variation for other traits to influence selection. We varied relatedness by initiating each mouse infection with either a single clone, to give relatively high relatedness, or with multiple clones, to give relatively low relatedness. In the relatively high relatedness treatment, each mouse is therefore infected with a single clone, which could be either the wild-type or the *lasR* mutant, and so cells only have the potential to interact with genetically identical cells, corresponding to a relatedness of *r* = 1 [19]. In contrast, in the relatively low relatedness treatment, each mouse is infected with multiple clones, allowing the potential for both the wild-type and the *lasR* mutant to be in the same mouse. In this case, cells have the potential to interact with both different and identical cells, corresponding to a relatedness of *r* < 1 [19]. For example, if there was an equal mixture of *lasR* and wild-type cells in a host, and both types were equally abundant in the population more generally, then this would correspond to a relatedness of *r* = 0.5 [19] The abundance in the population must be specified, because *r* depends on genetical similarity relative to the population.

We started the high relatedness treatment by infecting three mice with PA14, and three mice with the *lasR* mutant. After 24 h growth, we pooled the samples from these mice and randomly isolated five individual *P. aeruginosa* clones. Each of these clones would be either a wild-type or a *lasR* mutant. We grew each of these clones overnight separately in Luria Bertani (LB) broth at 37°C, shaking at 250 r.p.m., and used each of them to infect a single mouse. After 24 h growth, we harvested and sampled the bacteria in the same way, initiating five new mouse infections. In each round of growth, we therefore divided the population into five subpopulations, each housed within a mouse. The key points here are that in our high relatedness treatment: (i) each mouse is infected by only one clone, which will be either the wild-type or the *lasR* mutant, and so these two different types are not able to interact within mice; and (ii) global competition occurs between the bacteria from different mice, as when there is a higher growth rate within an individual mouse, then the bacteria from this mouse will contribute a higher proportion of the bacteria pooled from all the mice, and so they are more likely to be represented in the bacteria chosen to initiate the next passage.

In contrast, in our low relatedness treatment, we used multiple clones to initiate each mouse infection. In the first round, we infected three mice with a 50 : 50 mixture of PA14 and the *lasR* mutant. Then, in all subsequent rounds, we initiated each infection with an equal mixture (10^2^ each) of 10 clones. In order to calculate the relative frequency of *lasR* mutants after each round of infectivity, we also plated wound homogenates onto PIA containing gentamicin (100 μg ml^–1^). We were able to distinguish *lasR* mutants from PA14 on this media, because the mutants harbour a gentamicin resistance cassette. The key point here is that in our low relatedness treatment, each mouse is infected by a mixture of clones, which can be a mixture of both the wild-type and the *lasR* mutant, and so these two different types are able to interact within mice. This allows competition within hosts, where the *lasR* mutant could potentially exploit the extracellular factors produced by the wild-type, as well as between the bacteria from different mice.

We repeated this experiment three times, giving a total of six replicate selection lines (three at high relatedness and three at low). We repeated the selective regime for up to six passages through novel mice, with some selection lines being terminated earlier because either the wild-type or the *lasR* mutant had gone to fixation. Overall, our experiment involved the passaging of bacteria through a total of 191 mice. Our experimental design is analogous to previous *in vitro* experimental evolution studies examining how population structure and evolution influences the evolution of social traits such as QS [11–13,20,21], except that the subpopulations were grown in mice, not test tubes.

## 3. Results

**Figure 2.**
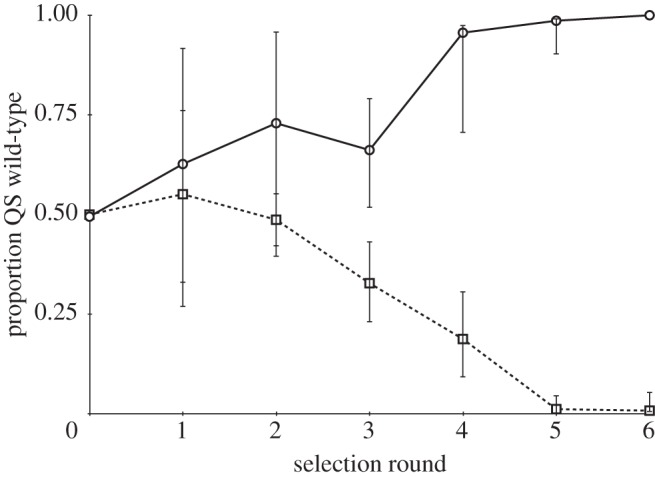
Quorum sensing is favoured by higher relatedness. The proportion of QS individuals (wild-type) is plotted against rounds of selection. Error bars represent the s.d. of three independent replicate selection lines per treatment. Circles denote high relatedness, whereas squares denote low relatedness.

**Figure 3.**
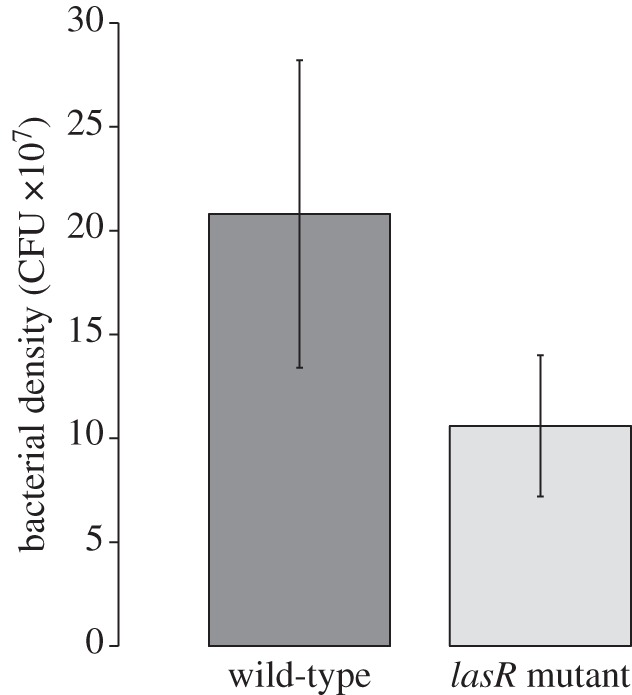
Bacterial growth in mouse burn wounds. Infections initiated with the QS wild-type grew to higher densities than those initiated with the *lasR* mutant. Bacterial density is the estimated number of colony-forming units per gram of tissue. Error bars represent the 95% CI.

We found that the QS wild-type was favoured under conditions of high relatedness, whereas the *lasR* (no response) mutant was favoured under conditions of low relatedness (figure 2; *F*_(1,4)_ = 284.9, *p* < 0.0001). Comparing mice that had been infected with only the QS wild-type or the *lasR* mutant, we found that the QS wild-type grew to significantly higher densities (figure 3; *F*_(1,66)_ = 4.3; *p* = 0.043). This result held irrespective of whether we analysed only the wound tissue, or the combined samples from both wound tissue and liver.

**Figure 4.**
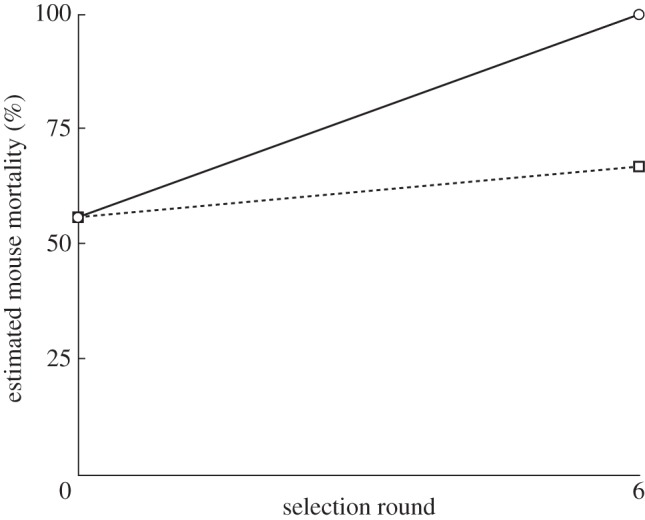
Kin selection and virulence. Shown is the predicted mouse mortality rate, 5 days after infection, at the start and end of our selection experiment. A higher relatedness favours QS, which facilitates bacterial growth and hence leads to higher virulence. Circles denote high relatedness, whereas squares denote low relatedness.

We then estimated the virulence consequences of variation in relatedness, by combining the results of this selection experiment with data that we have already collected on the mortality rate in infections of mice. Specifically, we have previously found that the percentage of mice that had died 5 days after infection was 100 per cent (9/9), 67 per cent (6/9) and 56 per cent (5/9) for infections initiated with the QS PA14 wild-type, the *lasR* mutant and a 50 : 50 mixture of the two, respectively [16]. Consequently, we estimate that high relatedness would lead to a greater virulence (100% mortality after 5 days) than low relatedness (67% mortality after 5 days; figure 4).

## 4. Discussion

We found that QS was favoured by a relatively high relatedness (figure 2). In the high relatedness treatment, the QS wild-type and *lasR* mutant occurred in different mice, and so the greater growth of the QS wild-type (figure 3) led to the wild-type increasing in frequency until it was fixed at 100 per cent. In contrast, in the low relatedness treatment, the QS wild-type and *lasR* mutant were able to co-exist in the same mice. When this happens, *lasR* mutants are able to exploit the extracellular factors produced by the QS wild-type, without paying the cost of producing them, and so the *lasR* mutants increase in frequency [16]. Put simply, conditions of high relatedness mean that cooperators interact with cooperators, and so cannot be exploited, whereas conditions of low relatedness mean that cooperators and cheats can interact, allowing cheats to exploit cooperators. Overall, these results provide clear support for kin selection theory, both as applied to QS and more generally [4,7,10,22]. Furthermore, by showing how relatedness influences selection on a specific molecular mechanism of pathogenesis, we have demonstrated how social evolution can shape selection on pathogen virulence (figure 4).

### (a) Relatedness and quorum sensing

Our experiment examined the consequences of variation in genetical relatedness for a single trait (whether or not to respond to QS), and not across the whole genome. We did this because we were interested in how that trait evolved, and so wished to remove noise due to selection on other traits. If we are interested in how a single trait evolves in response to relatedness, then the relatedness that matters, as defined in Hamilton's rule, is the genetical relatedness of that trait [7,19,23–25]. More specifically, relatedness is defined statistically as the genetical similarity between social partners relative to the rest of the population [19,26]. Consequently, in our experiment, a high relatedness means that hosts are infected with either the wild-type or the *lasR* mutant, such that cells will interact with identical cells, such that the *lasR* cells are not able to exploit the extracellular factors produced by the wild-type. In contrast, a low relatedness means that hosts can be infected by both the wild-type and the *lasR* mutant, such that the *lasR* cells are able to exploit the extracellular factors produced by the wild-type.

It is important to distinguish here between experiments with genetic manipulations at a single locus (in this case *lasR*) and more natural scenarios. In experiments such as we have carried out here, there is only genetic variation and hence the potential for relatedness to vary at the loci of interest, with no variation, and hence *r* = 0 at all other loci (relatedness is defined relative to the population, and so if you are as genetically similar to your social partner as to the whole population, then *r* = 0, even if you are genetically identical—this is analogous to why humans should not be expected to be especially altruistic to chimpanzees just because of the high similarity across our genomes [26]). While such manipulations are a bit artificial, they are experimentally useful, because it allows us to focus on selection on a single trait, such as QS.

In contrast, in natural scenarios, relatedness will be more similar across the whole genome [19]. A key point here is that while it is genetical relatedness at that locus that determines selection on a locus [7,23], as emphasized by selection on greenbeard genes [7,27,28], there is something special about when common ancestry causes genetic relatedness across the genome [19]. Common ancestry causes approximately the same relatedness across all alleles, and hence allows different parts of the genome to pull in the same direction to produce adaptations [19]. A potentially important complicating factor here, which does not arise in organisms typically used to study social evolution (such as insects and vertebrates), is the potential for horizontal gene transfer, and how it can cause relatedness to vary across the genome [8,29,30]. Measuring relatedness in natural populations of microbes, let alone if and how it varies across different social traits, remains a major task [15].

### (b) Relatedness, strain diversity and virulence

The prediction from our results—that higher relatedness will lead to higher virulence (figure 4)—is in the direction opposite to that from classical virulence theory. Numerous theoretical models have predicted that a lower relatedness between the parasites infecting a host (higher strain diversity) will lead to greater competition for host resources, and hence selects for a higher growth rate that leads to greater virulence [31–33]. We obtained the opposite result in our experiment, because a higher relatedness favoured greater cooperation between bacterial cells, which facilitates bacterial growth and hence leads to host mortality. This supports the predictions of theoretical models that have allowed for such cooperation [5,34,35], and is in agreement with previous empirical work on a bacterial phage [36,37].

A key difference here is that classical models assume that growth rate can be varied in response to the level of competition, whereas our results flow from the growth rate, depending upon the level of cooperation [5,38,39]. It is not that one group of models is wrong and the other correct, but rather that they focus on different types of social interaction, with different mechanisms of pathogenesis (public goods and cooperation versus restraint and a tragedy of the commons). In natural infections, there could be an interaction between these factors, with host mortality placing a cap on the growth rate that would be selected for at high relatedness [5], or even different relationships from other forms of social trait [39–41]. Within the specific context of our experiment, bacteria were passaged before host death, and so host death *per se* did not influence selection on QS, although theory suggests that the effects of host death on virulence can be relatively negligible compared with the effect of cooperation [5]. More generally, the majority of ‘virulence factors’, whose production are associated with virulence in bacterial infections, appear to be extracellular factors [9], and so we would expect that cooperation will be a driving factor in the virulence of bacterial infections.
